# Nanocomposites Based on Biodegradable Polymers

**DOI:** 10.3390/ma11050795

**Published:** 2018-05-15

**Authors:** Ilaria Armentano, Debora Puglia, Francesca Luzi, Carla Renata Arciola, Francesco Morena, Sabata Martino, Luigi Torre

**Affiliations:** 1 Department of Ecological and Biological Sciences, Tuscia University, 01100 Viterbo, Italy; 2 Civil and Environmental Engineering Department, Materials Engineering Center, University of Perugia, UdR INSTM, 05100 Terni, Italy; debora.puglia@unipg.it (D.P.); francesca.luzi@unipg.it (F.L.); luigi.torre@unipg.it (L.T.); 3 Research Unit on Implant Infections, Rizzoli Orthopaedic Institute, 40136 Bologna, Italy; carlarenata.arciola@ior.it; 4 Department of Experimental, Diagnostic and Specialty Medicine, University of Bologna, 40126 Bologna, Italy; 5 Department of Chemistry, Biology and Biotechnology, University of Perugia, 06100 Perugia, Italy; effemorena@gmail.com (F.M.); sabata.martino@unipg.it (S.M.)

**Keywords:** nanocomposite, biodegradable polymer, nanofiller

## Abstract

In the present review paper, our main results on nanocomposites based on biodegradable polymers (on a time scale from 2010 to 2018) are reported. We mainly focused our attention on commercial biodegradable polymers, which we mixed with different nanofillers and/or additives with the final aim of developing new materials with tunable specific properties. A wide list of nanofillers have been considered according to their shape, properties, and functionalization routes, and the results have been discussed looking at their roles on the basis of different adopted processing routes (solvent-based or melt-mixing processes). Two main application fields of nanocomposite based on biodegradable polymers have been considered: the specific interaction with stem cells in the regenerative medicine applications or as antimicrobial materials and the active role of selected nanofillers in food packaging applications have been critically revised, with the main aim of providing an overview of the authors’ contribution to the state of the art in the field of biodegradable polymeric nanocomposites.

## 1. Introduction

Environmental issues have resulted in significant industrial and academic research efforts aimed at developing “green materials,” based on raw materials derived from natural resources, as on biodegradable and biopolymer-based materials. One of the most promising biopolymers is polylactic acid (PLA), since it is obtained from agricultural products and it is biodegradable. Nanotechnology presents new opportunities to enhance material performance; of particular interest is the recently developed nanocomposite technology, because it permits the development of new polymeric materials with improved mechanical, thermal, electrical, and various other properties, in comparison with those of a virgin polymer [1,2,3,4,5]. Polymeric nanocomposites are a novel class of materials that can be regarded as a multiphase solid material, film or bulk, with one phase (the filler) that has one, two, or three dimensions in the nanometer range. Nanocomposites have attracted a lot of attention in academic research and in industry due to their unique properties compared to conventional composites, even at low filler content; these properties came from the nanometric size effect. A large variety of nanocomposites has been prepared by using different polymer matrices, and nanofillers. The nanofillers have strong reinforcing effects and studies have also shown their positive impact on barrier properties. However, most of the studies have been conducted with non-renewable inorganic fillers. Increasing environmental concerns have led to investigations of the potential use of renewable resources for specific applications, such as biomedical and packaging.

The aim of this review is to discuss and compare our recent papers on the design, development, and characterization of different nanocomposites based on biodegradable polymers, mainly focused on biomedical and packaging applications. Our results will also be compared with the scientific literature.

## 2. Materials

### 2.1. Biodegradable Polymers

Polymer nanocomposites with the main commercial polymer matrices have been developed and nano-scale filler dispersion has been achieved with varying degrees of success. Therefore bio-based nanocomposites and biodegradable nanocomposites have become a topic of interest in recent years and a number of suitable bio-based and biodegradable polymer matrices have been developed and their properties enhanced after the incorporation of different nanoscale materials. These systems include biopolymers from agricultural resources, such as polysaccharides and proteins, from biotechnology (as an example, poly(lactic acid), poly(hydroxyalkanoates) or biopolymers from petrochemical sources. e.g., PCL). All of them have been extensively reviewed in many papers, even recently [6], making reference to fundamental industrial sectors, such as food packaging [7] or the biomedical field [8]. The authors of the present work produced extensive work on this specific subject that can be found summarized in a few of their recent review papers that comprehensively discussed the role of polymeric matrices in the production of nanocomposites materials [1,2,3,4,5].

### 2.2. Nanofillers

**Figure 1:**
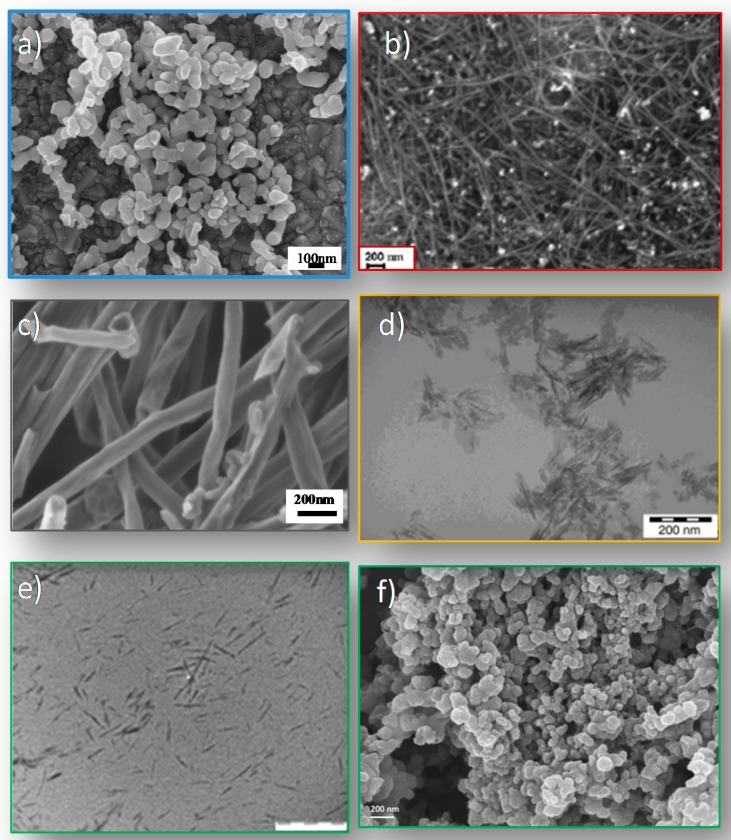
Electron microscopy images of the main used nanofillers: silver nanoparticles (**a**), single-walled carbon nanotubes (**b**), carbon nanofibers (**c**), nanohydroxyapatite (**d**), cellulose nanocrystals (**e**), and lignin nanoparticles (**f**). Adapted with permission from [9,10,11,12].

Different kind of nanofillers were used, both organic and inorganic materials. We can divide them into metallic nanoparticles, carbon nanostructures, nanohydroxyapatite, nanocellulose, and nanolignin. Figure 1 shows electron microscopy images of the main nanofillers used: silver nanoparticles (a), single-walled carbon nanotubes (b), carbon nanofibers (c), nanohydroxyapatite (d), cellulose nanocrystals (e) and nanolignin (f). A brief description of each nanomaterial, with an emphasis on the structure, properties, functionality and synthesis, is shown below.

#### 2.2.1. Silver Nanoparticles

Bulk silver is a well-known metal element, recognized for its high chemical stability, light sensitivity, bactericide ability, and catalytic properties (extensively employed in fabrication of coins, tableware, jewellery, medicine and photography), and for its broad antimicrobial activity. Silver nanoparticles (Ag NPs) are nanometric materials with unique optical and chemical properties, which can be finely modulated during synthesis by the right selection of the process parameters [13]. Ag NPs have gained considerable interest in recent decades because of their unique optical, electronic, catalytic, and antimicrobial properties, which differ significantly from those of bulk substances. These attractive properties of Ag NPs have permitted their potential use in several applications, such as catalysis, biosensing, drug delivery, and biomedical nanodevice fabrication, and they are dimension correlated. The large specific surface area and high fraction of surface atoms on silver nanoparticles (Ag NPs) lead to enhanced antibacterial activity compared to bulk silver metal.

The size and the shape of Ag NPs can be tuned appropriately. The literature presents several preparation methods to obtain various types of Ag NPs, mainly in terms of shape: spherical, rods, dog bones, prisms; and optical properties that can be classified as physical, chemical and biological methods. Synthesizing metallic NPs with different shapes is possible to date. Nanospheres, nanorods, and nanocups, etc. can be synthesized, and it is also possible to control and modulate the dimension of these nanoparticles. However, synthesizing silver NPs with uniform shapes and precise control of the nanometer size still remains a challenge, attracting several researchers worldwide. Ag NPs can be synthesized using various methods, such as chemical reduction [14], electrochemical [15], γ-radiation [16], laser ablation [17], solvothermal [18], hydrothermal [19], photochemical [20], sonochemical [21], and sputtering methods [22]. Ag NPs can be produced with various sizes and shapes depending on the fabrication method.

Silver nanoparticles absorb and scatter light with extraordinary efficiency; these unique optical properties originate from the collective oscillations of conduction electrons, which, when excited by electromagnetic radiation, are termed surface plasmon polariton resonances (SPPR) and it causes the absorption and scattering intensities of silver nanoparticles to be much higher than identically sized non-plasmonic nanoparticles. The optical properties are strictly correlated to the nanoparticles’ shape and dimension. Smaller nanospheres primarily absorb light and have optical absorption peaks near 400 nm, while increasing the dimension, increased scattering, and peaks that broaden and shift towards longer wavelengths occurred (known as red-shifting) [23]. The introduction of silver nanoparticles into various polymeric matrices induces antibacterial performance and modulates optical behaviour by extending the utility of NPs in material and device applications [24,25].

#### 2.2.2. Carbon Nanostructures

Nanocarbon materials such as fullerene, carbon nanofibers, carbon nanotubes and graphene have been considered interesting nanofillers in polymer nanocomposites. Since the discoveries of carbon nanotubes in 1991 [26], they have been extensively studied as another realization of the remarkable electronic properties of graphitic carbon. Carbon nanotubes may be schematized as a single graphene sheet rolled in a cylindrical way; they may be either multi-walled or single-walled. The latter, first clearly isolated in 1993 [27], have been studied more extensively and lend themselves better to theoretical analysis by virtue of their simpler structure. Single-walled carbon nanotubes (SWCNTs) can be described as individual graphene sheets “rolled up” and joined into cylindrical tubes. There are many possible way to join the edge of a graphene sheet and preserve the sp^2^ graphene structure. Graphene (G), a two-dimensional (2D) form of graphite, is a one-atom-thick planar sheet of sp^2^-bonded carbon atoms densely packed together in a honeycomb hexagonal lattice [28]. Graphene, with its covalently bonded 2D layers held together by van der Waals interactions, is in a sense the extreme 1D limit of a molecular crystal. A molecular crystal consists of a spatially periodic, extended array of individual molecules with covalent intramolecular interactions and van del Waals intermolecular interactions. Graphene shows high optical transmittance and high electrical conductivity. Carbon nanomaterials are now commonly grown via chemical vapour deposition. Much effort has gone into investigating the nucleation and growth processes and their kinetics [29]. Carbon nanostructures have attracted the attention of the scientific community for their extraordinary properties, including mechanical, electrical, thermal and optical. In recent years, many efforts have been dedicated to the incorporation of carbon nanostructures in polymer matrices, in order to modulate the polymer properties, mainly electrical and mechanical, and extend their applications [30,31,32].

#### 2.2.3. Nano-Hydroxyapatite

Hydroxyapatite (HAP, Ca_10_(PO_4_)_6_(OH)_2_) is the main constitute of the bone; it possesses excellent biocompatibility with bones, teeth, skin and muscles, both in vitro and in vivo. HAP promotes faster bone regeneration and direct bonding to regenerate bone without intermediate connective tissue, demonstrating good bioactivity and osteoconductivity, which permit extensive applications in orthopedics and dentistry [33]. Natural and synthetic HAP are currently applied in the biomedical field, as well as in clinical applications. HAP can be synthesized using chemicals containing calcium and phosphate ions [34]. However, during the last decade, natural materials have been shown to be an attractive source of HAP. Furthermore, to better mimic the mineral component and the microstructure of natural bone, nanohydroxyapatite has been synthesized. However, the main limitation on the use of HAP ceramics was their inherent brittleness and difficulty of processing [35]. To combine the osteoconductivity of calcium phosphates and good biodegradability of polyesters, polymer/ceramic composite scaffolds have been developed for bone tissue engineering either by direct mixing or by a biomimetic approach [36,37].

#### 2.2.4. Cellulose Nanocrystals

Cellulose is the most important polymer on Earth, and in the last year it has been increasing in use due to advantages such as rich resources, good thermal and chemical stability, high hydrophilicity and excellent biocompatibility. These fascinating properties have made cellulose a potential material for different applications in nano-drug delivery systems, biomedical applications and the food packaging field. Cellulose is produced from plants forming micro-fibrils, which in turn aggregate to form cellulose fibres. The fibre size can be disintegrated from micro-fibrils to nanofibers by physical and chemical methods. The hydrolysis treatment with sulfuric acid to prepare cellulose nanocrystals has been extensively investigated and appeared to be the most effective method.

Cellulose nanofibers (CNF) can be a new building block for renewable smart materials. CNFs have excellent mechanical strength, dimensional stability, thermal stability and good optical properties on top of their renewable behaviour. The presence of many hydroxyl groups along the cellulose chain results in the formation of a network of intra- and intermolecular bonds [38]. In addition, a network of van der Waals connections is established between the chain layers. These linked networks, and the stiffness of the glycosidic bond, allow for the construction of ordered crystalline structures, 2–20 nm in width and up to a few microns in length. These crystalline domains are interspersed with amorphous regions in the fibril structure. Cellulose nanocrystals occur as high-aspect ratio rod-like nanocrystals. Their geometrical dimensions depend on the origin of the cellulosic substrate and hydrolysis conditions. The impressive mechanical properties make cellulose crystal an ideal candidate for the processing of reinforced polymer composites. Its Young’s modulus is much higher than for glass fibres, around 70 GPa. It is similar to Kevlar (60–125 GPa) and potentially stronger than steel (200–220 GPa), and in general it depends on the extraction methods. CNCs show great applications in green material science and biomedical applications [39,40].

#### 2.2.5. Lignin

Lignin, the second most abundant natural aromatic polymer on earth, is a three-dimensional, highly cross-linked macromolecule composed of three types of substituted phenols yielding a vast number of functional groups and linkages and variable chemistry depending on its origin [41]. It should be considered that important industrial organic chemicals such as hydrocarbons, alcohols, polyols, ketones, acids and phenol derivatives can be easily obtained by chemical processing of lignin, while new potential lignin applications, such as (a) lignin valorisation for bio-fuels and energy production, (b) lignin exploitation toward high molecular mass applications like polymers (e.g., polyurethane foams), wood adhesives and carbon fibres and (c) lignin utilization for the production of polymer building blocks, aromatic monomers including benzene, toluene, and xylene, phenol and vanillin, have recently been considered.

Lignin can be considered an alternative lignocellulosic source to be incorporated in polymeric systems. Studies on the use of large size lignin in thermoset, thermoplastic, or elastomer-based composites have been recently discussed and reviewed by Thakur et al. [42]. More recently, insight into lignin’s molecular structure has encouraged chemists to develop methods for the precipitation of lignin nanoparticles, towards the ultimate goal of valorising lignin to produce value-added products. In recent years, different lignin nanoparticles (LNPs) from various resources were synthesized by different chemical/physical approaches [43,44]. However, the lignin propensity to self-aggregate negatively influences its dispersion in many composite formulations; therefore, many methods were attempted and considered to achieve good dispersion of lignin particles in a biodegradable matrix [45].

## 3. Methods

Several strategies have been considered to develop nanocomposites based on biodegradable polymers, and can mainly be divided into solvent-based and melt-mixing methods. The process technology is an important step in the final nanocomposite development since it can affect the dispersion grade of the nanofillers in the polymers and hence the functional properties and the micro- or nanostructure of films or 3D structures. The homogeneous dispersion of the nanofillers within a continuous polymeric matrix is a key step to benefiting from their outstanding properties. The main criteria for the selection of the appropriate process technology for nanocomposite manufacture include the desired product geometry, the performance needed, and the cost and the ease of manufacture. The different processing techniques in general do not yield equivalent results.

### 3.1. Solvent-Based Process

Solvent casting is one of the simplest methods to develop nanocomposites. The main points in the method are:

- selection of the solvent: the solvent has to permit a complete dissolution of the selected polymer and, at the same time, provide a good dispersion of the nanostructures in the polymer matrix;
- the relative concentration between polymer and solvent, since it can affect the viscosity of the solution and the evaporation rate;
- the external conditions (pressure, temperature, and humidity) during the solvent evaporation, since they can affect the properties of the nanocomposites and the surface morphology.

An important point to consider is the complete evaporation of the solvent before using the final nanocomposite, since it can be toxic and affects the properties. In industry and also in scientific research laboratories, the use of organic solvents such as chloroform is usually avoided; hence the possibility of using a water-soluble polymer is an important strategy. However, the solvent-based process limits the number of polymer matrices that can be used and this procedure is both non-industrial and not economically viable. It should be used for niche applications. In our research activities we used the solvent-based approach mainly when we dispersed a small amount of synthesized nanoparticles in a polymer soluble in organic solvent, and also in the case of solvent-stable soluble nanoparticles [9,46,47,48,49,50,51,52,53,54,55,56].

### 3.2. Melt Mixing

Melt-compounding methods, such as extrusion or injection moulding, are commonly used to process thermoplastic polymers. They are “green” (solvent-free), and industrially and economically viable. The production of the final nanocomposites is optimized in relation to temperature, pressure, and moulding time. The polymer industry has been using extrusion for decades as a high-volume manufacturing process to produce many different items such as tubes, frames, plastic sheets and films [57]. However, during these processes high temperatures will be used, so thermal degradation of the polymers should be avoided. In spite of high operating temperatures and pressures, as well as high shear rates, the processing of polymers by extrusion is a widely used process in industry because it is a continuous, quick, simple and versatile operation for transforming raw ingredients into finished products.

Furthermore, the method is environmentally benign due to the absence of organic solvents, and is compatible with current industrial processes [58,59]. Each polymer system requires a special set of processing conditions to be formed, based on the processing efficiency and product properties desired.

## 4. Biodegradation

Biodegradation properties of nanocomposites based on biodegradable polymer are of crucial importance in new material development. Different biodegradation conditions can be considered: hydrolytic, composting, enzymatic, according to the final applications and the post-use of the new developed materials.

The degradation of a polymer is a complex phenomenon that involves different parts; in particular, in the case of PLA compost degradation we can observe four main phenomena, namely water absorption, ester cleavage and formation of oligomer fragments, solubilisation of oligomer fragments, and finally diffusion of soluble oligomers by bacteria [60]. The initial step of PLA disintegration is surface hydrolysis; the polymer is disintegrated in low molecular weight species. Molecular weight variations are an indication of the degradation rate of the polymers and give information about when the main fragmentation occurs in a polymer. Firstly, the polymer amorphous phase is attacked by microorganisms at the initial stage of the disintegration process, which leads to a loss of transparency. The addition of nanostructures in a biodegradable polymer matrix was found to affect all the nanocomposite properties, in particular the degradation rate, and this effect is dependent on the type of filler. It was also found that clays can influence the polymer bacterial degradation depending on their chemical structure and the affinity of the bacterium towards clay. In composting, the addition of nanoclays was found to increase the PLA degradation rate because of the presence of hydroxyl groups belonging to the silicate layers of these clays [60,61]. Also, cellulose nanocrystals (CNCs) increased the disintegrability rate of PLA, due to the hydrophilic nature of nanocellulose [62].

In the case of carbon nanostructures, to date, the impact of CNT inclusion on the stability of biopolymers has been restricted to a few studies, most of which involve enzymatic decomposition. In our previous paper we studied the in vitro degradation of PLGA and PLGA nanocomposite films based on SWCNTs and oxidised SWCNTs, by analysing the role of the content and functionalization. We found that all PLGA films were degraded by hydrolytic degradation and a different mechanism was observed in the case of functionalized SWCNTs with respect to pristine material [63]. The incorporation of SWCNTs increases the dimensional stability of the polymeric samples but they do not seem to significantly modify the kinetics and the mechanism of the hydrolytic erosion with respect to the neat PLGA. The presence of carboxylic groups in functionalized SWNTs-COOH accelerated the hydrolytic degradation of the PLGA matrix and the weight loss of the nanocomposites.

Singh et al. showed that a 1% mass loading of CNTs dispersed in PLA accelerated the enzymatic decomposition rate of PLA using Proteinase K [64]. This accelerated decomposition rate was attributed to a number of possible reasons including an increase in amorphous zones that were more susceptible to enzymatic hydrolysis due to the functionalization or potentially higher enzyme binding to the CNTs in the polymer nanocomposite substrate.

In contrast, other studies have shown that MWCNTs reduce the polymer biodegradation rate [65,66]. Recently, Goodwin et al. have explored the effect of CNT loading and type on PCL biodegradation in the presence of *P. aeruginosa* [67]. The rate of PCL matrix biodegradation decreased systematically as the CNT loading increased from 0.1 to 10% *w/w*. The addition of even a low CNT loading (<1% *w*/*w*) caused the CNT/PCL biodegradation rate constant to decrease by more than 50%. Similar trends in biodegradation rate were observed for both pristine and oxidized multiwall CNTs embedded in PCL. During PCL matrix biodegradation, CNT accumulation was observed at the surface of CNT/PCL nanocomposites and single particle inductively coupled-mass spectrometry experiments revealed no measurable CNT release to the culture fluid [67].

## 5. Biomedical Applications

The new biodegradable nanocomposites with modulated bulk and surface properties have attracted much attention in tissue engineering and regenerative medicine applications. Tissue engineering approaches require proper stem cell types and a selected biocompatible material that allowed the differentiation of stem cells towards the tissue-specific lineage [68,69]. Alternatively, primary cells can be used [70].

### 5.1. Stem Cells

Stem cells are a valuable subset of cells endowed by the self-renewal and pluri/multipotential properties [71]. The self-renewal property allows stem cells to provide a reservoir that persists over the tissue/organ life within a special microenvironment where the stem cells reside (niche). Here, stem cells could divide in an asymmetric way to give rise to two daughter cells, one that remains a stem cell and one committed cell, or two stem cell daughters (symmetric division) [72,73,74,75,76]. Committed cells, upon specific molecular signals, generate a tissue-specific differentiated cell linage and maintain the tissue homeostasis [77,78,79]. Stem cells are classified based on their origin and biological properties. Embryonic stem cells (ESCs) are isolated from the inner cell mass of blastocysts, have self-renewal property and are pluripotent since they are capable of generating all types of cells present in adult tissues as well as all types of adult stem cells [80,81].

Adult stem cells (ASCs) are created during development and endure within the niche of adult tissues/organs; they have self-renewal and multipotent properties since they are capable to generate a restricted differentiation lineage [82,83,84,85]. Notably, ASCs maintain the characteristics of their embryonic origin. Thus, ASCs isolated from the liver, lungs and pancreas have endodermal features; stem cells isolated from adult bone marrow and muscle have mesodermal characteristics; ASCs from the central nervous system or epidermis have ectodermal features [86,87]. Within the tissues, ASCs are mainly involved in maintaining the tissues’ homeostasis. Induced pluripotent stem (iPSCs) cells are stem cells generated in vitro from fibroblasts (somatic differentiated cells) by the addition of four genes, Oct3/4, Sox2, c-Myc and Klf4. iPSCs have ESCs characteristics. They have self-renewal property and are pluripotent [88].

For several years, ESCs, ASCs and iPSCs have been evaluated as potential cell sources for tissue engineering applications. Many reports have explored the effects of polymers and gels with different formulations or forms (fibrous, films and three-dimensional structure) on stem cell fat. Some examples are reported in Table 1.

### 5.2. Primary Cell Lines

Primary cells are isolated from living tissues/organs cultured in vitro under conventional growth culture conditions. These cells are representative of the tissue or of a selected part of the tissue from which they are derived. For instance, fibroblasts have the features of the connective tissue from which they are isolated. These cells are differentiated cells, with a large, flat and elongated morphology, and produce one of the main components of the extracellular matrix. Cultures of primary cells can be used as a suitable model for preclinical studies. These include the combination with biomaterials for establishing ex vivo models of tissue engineering [70].

### 5.3. Bacteria

**Figure 2:**
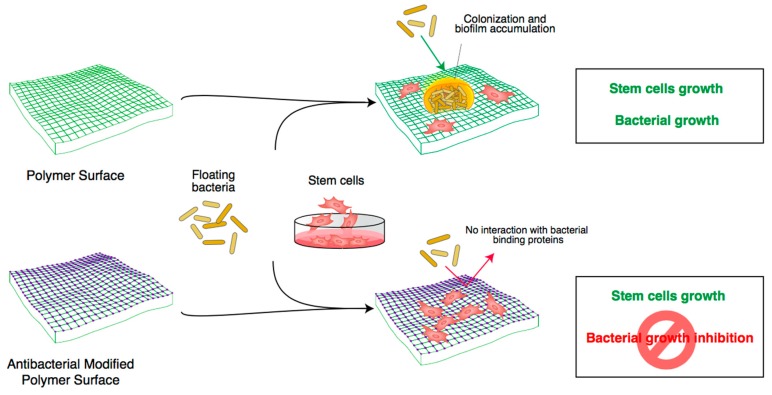
Cartoon shows how advancements in engineering materials combat bacteria biofilm formation and in turn permit the growth and differentiation of stem/primary cells for biomedical purposes. The “purple” square represents a basic polymer with specific antibacterial modifications to block bacterial growth and the biofilm accumulation and allow the growth and differentiation of stem cells. The “green” square represents a basic polymer (without antibacterial modifications) that is suitable for stem cell growth/differentiation and floating bacteria.

Bacteria are microscopic prokaryotic organisms of single cell with a simple internal structure, and prosper in various environments (e.g., soil, ocean, inside the human gut) [10,111]. Compared to eukaryotic cells, they lack organelles. In fact, they have a simple internal structure, where reside proteins and molecules necessary for the bacteria life including DNA [10,111]. The cell bacteria are encircled by two protective layers consisting of an outer cell layer and an inner cell membrane. In some cases a third external protective layer (capsule) has been described [112]. Bacteria are classified based on the nature of the outer membrane, the morphology and finally by genome. The latter classification considers the evolutionary relationships between bacteria. This phylogenetic classification became possible with the advent of next-generation sequencing that allowed for the automatic sequencing of nucleotides in both DNA and RNA molecules. The universal presence in all living cells of ribosomes permits the comparison of ribosomal RNA sequences [113]. A more general classification is based on the molecular composition of the outer membrane. The Gram test allows us to discriminate bacteria into Gram positive, if after the test they are stained with a purple dye called crystal violet, or Gram negative if after the test the dye is easily washed away during the staining process. *Escherichia coli* (*E. coli*) is a Gram-negative bacterium and *Streptococcus pneumoniae* is a Gram-positive bacterium. The relationship between humans and bacteria is quite complex. In some cases, they are critical for digestion (commensal bacteria) or for modification of selected nutrients (e.g., milk into yogurt). In other cases, they are devastating for tissues/organs, causing disease (pathogen bacteria) [114]. The discovery of antibiotics and vaccines, together with the use of hygiene, has drastically reduced mortality related to bacterial infections, and allowed the resolution of many diseases caused by some pathogenic bacteria [115,116]. Unfortunately, the so-called “antibiotic golden age” is now threatened by the occurrence of an increasing number of antibiotic-resistant bacteria types that generate a chronic status of the disease and make it difficult to eradicate infections [117,118]. During the last two decades, many research groups have progressively revealed the presence of bacterial communities that are protected by a surface biofilm. They showed that these bacteria communities are prevalent in all types of natural environments compared to individualized, planktonic bacteria [119]. Recent studies have shown that biofilms are composed of a self-produced polysaccharide matrix and have specific biological properties that save bacteria from the antibiotic’s toxicity [120]. The first case of the correlation of the biofilms and the persistence of the infections was revealed by the ability of *Pseudomonas aeruginosa* to colonize the lungs of cystic fibrosis patients [11,120]. After that, other researchers confirmed the role of biofilms in the pathology caused by bacterial infections [120]. It has been demonstrated that infection in patients with implanted medical devices is a consequence of the growth of microorganisms that adhere to and colonize the device [120]. With the intent to protect devices from bacterial infections, researchers are developing antibacterial coatings [12,121]. The aim is to develop a material with appropriate modifications that combats the bacterial infection and permits the cell/stem cell cultures (Figure 2). It was shown that the surface topography and roughness have a great influence on the attachment of bacteria to a material surface. Moreover, the attached bacteria and the nucleating biofilm may be inhibited by both surface nanotopography and surface chemistry [122,123,124]. Figure 2 shows the general approach and the advantage of polymers with peculiar modifications such as addition of chemical cues that have antibacterial activity. In fact, only antibacterial modified polymers became safe and suitable for stem cell culture [101,103].

### 5.4. Nanocomposite Interaction with Biological Entities

As described above, we have developed different kinds of nanocomposite materials, based on biodegradable polymers, in order to modulate electrical, thermal, morphological, surface and mechanical properties, upgrade the structural and functional properties of synthetic biopolymers through the introduction of organic and inorganic nanofillers, and study the effect of these properties modulation on specific biological entities such as stem cells [9,46,47,48,49,50,51,52,53,54,55,56]. The overall studies have been conducted by culturing the stem cells on polymer films under growth culture condition without the use of additional inducible molecules. We noted that either on a biodegradable polymer with fibrous structure and tri-dimensional (3D) microstructure or on a polymer with film form, stem cells interact by maintaining proliferation and differentiation properties. However, depending on the polymer structure and on the nanocomposite, we observed a different stem cell response [9,46,47,48,49,50,51,52,53,54,55,56].

#### 5.4.1. Binary Conductive Nanocomposites

SWCNTs and MWCNTs were used mainly to modulate the electrical and dielectrical properties in biodegradable polymer matrices, as PLLA. We found that pristine polymers show an insulator behaviour, with a dc electrical conductivity (σ) of around 10^−16^ S · cm^−1^. A small addition of MWCNTs (0.3 wt %) to PLLA yields a drastic raise in σ. In order to obtain a conductive nanocomposite, carbon nanotubes must form a three-dimensional conductive network in the polymer, above a critical concentration, known as the percolation threshold, where a transition from non-conducting to conducting state occurs. This threshold depends on the nanotube dispersion, as well as on the nanofiller aspect ratio, lowering the percolation threshold with the increase of aspect ratio [46]. The addition of carbon nanotubes to a polymer matrix increases thermal and electrical conducting areas (higher loads result in an increase in the probability of having more electroactive sites). Electrical behaviour and surface topography at micro- and nano-scale influence the communication between stem cells and polymer nanocomposites. We found that this active interaction between stem cells and nanocomposites is stem cell type-specific. Culturing human umbilical cord matrix stem cells (hUCMSCs) on PLLA polymer film and on PLLA/Multi-Walled Carbon Nanotubes (MWCNTs) nanocomposite films, we confirmed the ability of the PLLA to interact with stem cells and activate a specific biological response. In fact, we showed that the active interaction between hUCMSCs and PLLA and PLLA/MWCNTs nanocomposite films results in the stem cell assembly as a spheroid conformation. Interestingly we observed that the spheroids directly respond to a tunable surface and the bulk properties (electric, dielectric and thermal) of plain and nanocomposite PLLA films by triggering a mechanotransduction axis and in turn directing a selected stem cell fate transition. In fact, the presence and content of MWCNTs within the PLLA polymer steered the stem cell fate toward a Primitive Endoderm-like phenotype on PLLA/MWCNTs instead of an Epiblast-like phenotype on PLLA polymer [32,46].

Different behaviour was observed in the case of human bone marrow mesenchymal stem cells (hBM-MSCs) seeded in the same films. hBM-MSCs maintained a star shape and elongated configuration [46]. Our work represents a pioneering effort to create a stem cell/material interface that can model the stem cell fate transition under growth culture conditions [32]. The electroactive surface could efficiently improve the intracellular calcium level and then enhance myoblasts differentiation. Thus, conductive nanomaterials such as carbon nanotubes and gold nanoparticles have been used to prepare conductive nanocomposites for muscle tissue regeneration [125,126,127]. These studies demonstrated that a conductive material surface could efficiently guide myoblast proliferation and myogenic differentiation [125,126,127,128]. Recently Du et al. developed highly elastomeric, conductive and biodegradable poly(citric acid-octanediol-polyethylene glycol)(PCE)-graphene (PCEG) nanocomposites for the first time, demonstrating their applications in myogenic differentiation and guiding skeletal muscle tissue regeneration. The myoblast proliferation and myogenic differentiation were significantly improved by PCEG nanocomposite. Significantly high in vivo biocompatibility of PCEG nanocomposites was observed when implanted in the subcutaneous tissue of rats for four weeks [129]. When introducing the individual nanomaterial into a polymer matrix, it is important to achieve an adequate dispersion in the polymer matrix, as well as strong interfacial interactions between the nanomaterial and the polymer, in order to improve the load transfer across the nanofiller–polymer matrix interface. The functionalization of nanofillers has been considered one of the best approaches to prevent the aggregation due to strong van der Waals forces. In fact, in the case of CNTs the functionalization is a prerequisite in order to take advantage of most of the properties that enable facile fabrication of novel nanomaterials and nanodevices. Most of the functionalization approaches developed at present can be categorized into two categories: covalent and non-covalent functionalization. The surface functionalization of CNTs, as carboxylic acid, was used in our group to increase compatibility between PLLA and nanostructures, in order to improve the interface between polymer and CNTs and consequently the mechanical properties, since these properties have a strong effect on the cell properties and function [47].

#### 5.4.2. Ternary Conductive Nanocomposite

Multifunctional ternary films were also developed by the combination of SWCNTs and silver nanoparticles with a biodegradable polymer matrix, as the poly(ε-caprolactone) (PCL) polymer matrix by a solvent cast process. Nanostructural synergistic effects were evaluated. Results showed that the PCL/Ag nanocomposites exhibited poor electrical properties, while in PCL/Ag/SWCNTs ternary films higher values of conductivity were measured compared to both binary nanocomposites, indicating that Ag particles facilitate the formation of conductive pathways in the presence of SWCNTs, acting as conductive bridges among nanotube bundles and facilitating electron transfer. Both binary and ternary systems showed biocompatibility with respect to human bone marrow derived mesenchymal stem cells (hBM-MSCs) and antibacterial characteristic due to silver nanoparticle introduction. The suitability of these conductive nanocomposite films as a potential support for primary hBM-MSCs was demonstrated, showing comparable viability and cell–material interaction in the culture period [9].

#### 5.4.3. Porous Nanocomposites

Porous biodegradable nanocomposite materials are ideal candidates as scaffolds for the tissue engineering approach. Hence we decide to develop 3D structures using different methods such as electrospinning, solvent casting particulate leaching process, and thermal induced phase separation (TIPS) process [48].

Electrospinning of bioresorbable polymers is a promising and valuable technique to develop porous membranes. To improve its potential applications, the addition of specific fillers has been considered. In one paper we reported the fabrication of electrospun poly(l-lactic acid)/Ca-deficient hydroxyapatite (PLLA/d-HAP) mats; the content of nanosized d-HAP ranged between 1 and 8 wt %. All samples consisted of micrometric and submicrometric fibres, comprising 2D voids of 8 and 13 µm for PLLA and PLLA/d-HAP mats, respectively [36]. The dispersion of 1 or 8 wt % of HAP to the polymer changed the mechanical properties of the pristine PLLA materials, but the architecture remained similar [36,37]. Dynamic mechanical properties decreased with the increase of the HAP [37]. We used these mats to investigate the response of three different stem cell type: hBM-MSCs, murine-induced pluripotent stem cells (iPSCs) and murine embryonic stem cells (ESCs). We demonstrated that after three weeks of culture on PLLA/d-HAP, all stem cells expressed osteogenic markers such as BGLAP, BMP-2, RUNX-2 genes and the deposition of bone matrix proteins (decorin, fibronectin, osteocalcin, osteonectin, osteopontin, type I collagen, and type III collagen) in the absence of soluble osteogenic factors [37]. Conversely, multipotent and pluripotent stem cells maintain stemness on neat PLLA. We discussed the absence of osteogenic differentiation in stem cells cultured on neat PLLA polymer as a direct consequence of the dispersion of the different amounts of d-HAP into PLLA and in turn of the different levels of the stiffness [37]. Electrospun membranes permit us to simulate the extracellular matrix, but do not create the high pore structure necessary for cell migration. In order to induce cell migration and penetration, higher pores are needed, with defined morphology and good interconnectivity. The TIPS process is a valuable method to develop polymeric and nanocomposite scaffolds with pore diameter ranging from 30 to 150 μm and interconnected. Ternary systems by the TIPS process with nanoHAP and SWCNTs in PLLA polymer matrix with a porous structure were developed as a final result of a European project where Italy, Spain and Switzerland collaborated to develop a new biodegradable biomaterial that permits bone regeneration by specific factors in in vitro and in vivo conditions [49].

#### 5.4.4. Composites and Nanocomposites Based on Organic Fillers

Organic fillers as keratin and cellulose nanocrystals were also used as micro and nanofillers in biodegradable polymer matrix, both in 2D film and 3D structures. The formation of a new generation of hybrid composites was reported using bio-based fillers as CNCs with nano-dimension or keratin with micro-dimension. In one paper we aimed at modulating the mechanical, thermal and biocompatibility properties of poly(vinyl alcohol) (PVA) by a combination of cellulose nanocrystals and poly(d,l-lactide-*co*-glycolide) (PLGA) nanoparticles (NPs) loaded with bovine serum albumin fluorescein isothiocynate conjugate (FITC-BSA) [50]. Our approach aims to extend the use of biodegradable polymer NPs as drug delivery systems based on their encapsulation into PVA/cellulose nanocrystal bio-nanocomposites (PVA/CNC), thus offering a combination of two nanotechnology approaches in a unique system. These new systems combine the biocompatibility and high mechanical response of the PVA/CNC systems with the protein control release due to polymeric PLGA nanoparticles’ introduction. We used bone marrow mesenchymal stem cells to evaluate the biocompatibility of the nanocomposite films based on PVA (binary and ternary) and to explore the release of NPs and fluorescent BSA protein. Our results demonstrated that stem cells take easily up NPs released either in the culture medium by the binary films following the rapid PVA dissolution, or by ternary films through direct contact modality (stem cellular membranes/films). The obtained results demonstrate the possibility of creating a system able to release specific drugs thanks to the presence of PLGA polymeric nanoparticles, to interact with a selected biological system, such as bone marrow stem cells, and to control and modulate its mechanical properties in the presence of nanocellulose crystal dispersion. The effect of chemico-physical, topographical and mechanical properties of a biomaterial on the induction of stem cell differentiation [9,37,46,69] as well as on the preservation of stem cell status is well known in the scientific literature [130]. Stem cells’ ability to convert mechanical cues into biochemical signals and modulate their fate is a relatively recent finding in scientific research [131].

Keratins were also used as bio-based fillers in synthetic biodegradable polymers. We used keratins extracted from Merino wool (KM) and Brown Alpaca fibres (KA) as fillers in poly(l-lactic) acid based biocomposites and the performance of the developed materials was compared with commercial hydrolysed keratins (KH)-based biocomposites. The results confirmed that the surface morphologies of biocomposites revealed specific round-like surface topography of different microsized keratin particles in different weight contents, e.g., the analysis of bulk morphologies confirmed a phase adhesion strictly dependent on the keratin source. Transparency and thermal responses were significantly affected by the presence of the different keratins and their interaction with the PLLA matrix. Mechanical characterization by tensile test analysis underlined the possibility of modulating PLLA properties, selecting the keratin type and content in order to positively influence the elastic and/or plastic response. The presence of keratins also influenced the protein adsorption; moreover, PLLA and PLLA biocomposites based on different kinds of keratins supported the culture of hBM-MSCs. The presence and dispersion of keratin in the PLLA polymer enhances the interactions between human cells and polymeric membranes, as well as cell proliferation. These biocomposites might be promising materials for medical applications and the combination of human bone marrow mesenchymal stem cells with a biocomposite could open up new perspectives for the treatment of skin wounds [51,52,53].

#### 5.4.5. Nanocomposite Surface Properties

Modulating the nanotopography of the substrate, we demonstrated that stem cells follow the geometry and, in particular conditions, respond by activating a specific differentiation lineage. The interaction between stem cells and biomaterials with nanoscale topography represents a main route in the roadmap for tissue engineering-based strategies. In particular, we showed the promising prospects offered by the radiofrequency plasma processes on polymeric substrates by using polystyrene (PS) films as a reference substrate and developing specific micropatterned grooves and nanostructured roughness by using oxygen plasma treatment coupled with a mask [54,55]. We observed an improved wettability, roughness and etching rate by increasing the power supply and treatment time. Uniform and patterned bi-layer films show regular surface morphology and uniform chemical properties, with a water contact angle of 77°, a surface energy of 51.15 mN and good stability in physiological conditions. Nanoindentation measurements revealed a decrease in the bi-layer friction coefficient from 0.76 of PS to 0.17, highlighting the improvement of the nanomechanical properties of the novel developed system. Interaction with human bone marrow mesenchymal stem cells demonstrates that uniform and patterned PS-based films are biocompatible surfaces and that groove patterned substrates induce stem cell alignment and elongation [56,57].

In a recent work we have explored the effects of the interaction between the PLLA polymer film and human adult adipose stem cells (hASCs), focusing on the events correlating the materials’ surface characteristics and the cells’ plasma membrane. hASCs were seeded on films of pristine PLLA polymer and on a PLLA surface modified by the radiofrequency plasma method under oxygen flow (PLLA + O_2_). Comparative experiments were performed using hBM-MSCs and hUCMSCs. After treatment with oxygen plasma, the surface of PLLA films became hydrophilic, whereas the bulk properties were not affected. hASCs cultured on pristine PLLA polymer films acquired a spheroid conformation. On the contrary, hASCs seeded on PLLA + O_2_ film surface maintained the fibroblast-like morphology typically observed on tissue culture polystyrene. This suggests that the surface hydrophilicity is involved in the acquisition of the spheroid conformation. The oxygen treatment had no effects on hBM-MSC and hUCMSC cultures and both stem cells maintained the same shape observed on PLLA films. This different behaviour suggests that the biomaterial interaction is specific to stem cells [56].

#### 5.4.6. Antibacterial Nanocomposites

The incorporation of antimicrobial fillers in the biodegradable polymer matrix may permit the development of new multifunctional nanostructured materials with a good antibacterial response, also at very low nanoparticle concentration. With this purpose in mind, we developed a nanocomposite based on silver nanoparticles and synthetic biodegradable polymers, such as PLGA and PCL, by the solvent casting method. We evaluated the effect of different content of Ag NPs in terms of thermal, morphological, mechanical, surface and degradation properties of the polymer matrix, but also in terms of interaction with biological entities, evaluating the bacterial growth and also stem cell interaction. Furthermore the Ag+ release was monitored and correlated with the antibacterial properties. The results showed that, by controlling the nanoparticle content, tunable degradation and antibacterial action can be achieved [132,133,134,135]. The stem cell interaction is important in order to evaluate the effect of the nanoparticles in the nanocomposite on the biocompatible properties. The main issue when developing antibacterial polymeric nanocomposite materials is to reduce the bacterial growth without negatively affecting the cell vitality. Moreover, the surface properties were used as a valuable tool to modulate the first part of the material that came into contact with the biological entities, and can induce an antibacterial performance without changing the bulk properties. In particular, in our experiments we combined nanocomposites with the surface modification method. We developed nanocomposites based on a PLGA polymer matrix and different content of Ag NPs; then the surface of the PLGA polymer and nanocomposite was modified by oxygen plasma treatment by using a radiofrequency reactor that permits detailed control of the process parameters in terms of pressure, gas flow, power supply and bias voltage. Results showed that the PLGA surface becomes hydrophilic after oxygen treatment and its roughness increases over the treatment time. Our data confirm that surface modification treatment by the plasma method and Ag NPs introduction have a dominant influence on bacteria adhesion and growth [131].

## 6. Packaging Applications

In the last few decades, growing concern about the environmental contamination/pollution due to the high impact of plastic waste has led industry and academic researchers to develop sustainable and green polymers extracted from natural resources. In this context, biodegradable polymers have received much attention with respect to non-degradable polymers based on petroleum sources. Indeed, the use of green polymers represents a valid alternative for the food packaging sector. Packaging materials, in fact, represent the highest source of environmental pollution. Recent papers reviewed the use of green polymers combined with lignocellulosic nanoparticles, metal nanoparticles and some active ingredients in food packaging applications.

The food packaging sector represents one of the most important areas where nanotechnology has gained an important and innovative role for the development of packaging products. In detail, nanotechnologies have emerged to sustain and promote innovation in the packaging, production and preservation of foods to improve the shelf life of food products by modulating the polymeric characteristics [136]. Finally, multifunctional nanocomposites with structurally and functionally modulated properties, obtained by combining reinforcement phases as micro- and nanostructured lignocellulosic materials with antimicrobial agents (essential oils, natural extracts and metal nanoparticles), have been investigated.

The use of a nanocomposite approach guarantees improvement in the characteristic properties of polymeric matrices; specifically, nanofiller and active additives modulate the mechanical performance, e.g. mechanical strength, chemical stability, thermal stability, water vapour and gas (carbon dioxide and oxygen) barrier properties, recyclability, biodegradability, optical properties, antifungal and antimicrobial/active activity [136,137,138]. In this framework, we can identify polylactic acid (PLA) as one of the most used and investigated polymers in the food packaging sector. PLA is approved by the U.S. Food and Drug Administration (FDA) as a food contact material, often applied as packaging for some short shelf-life applications [139]. Nevertheless, PLA shows some limitations, such as poor thermal, mechanical and barrier properties, which are relevant in the food packaging sector.

As a consequence, several strategies have been considered to enhance the weak properties of this specific matrix. In particular, blending PLA with other biodegradable polymers or synthetic matrices represents one of the most effective methods to obtain new properties necessary for specific final-use applications [138]. For example, blending PLA with poly(hydroxybutyrate) (PHB) or poly(butylene succinate) (PBS) leads to polymeric materials with improved and interesting thermal, physical and mechanical characteristics when compared to neat PLA [59,136,140]. With the main aim of improving the mechanical and antimicrobial response of neat matrix, PLA-based composites loaded with microcrystalline cellulose (MCC) and silver nanoparticles (AgNPs) were also combined to demonstrate the advantages of a multifunctional system approach [141]. Fortunati et al. demonstrated the synergic effect of MCC and AgNPs on the final properties of PLA-based composites [141]: the results of mechanical characterization confirmed that MCC acted as a reinforcement phase in PLA matrix, while AgNPs induced bactericidal activity against Gram-negative bacteria (*Escherichia coli*) and Gram-positive bacteria (*Staphylococcus aureus*). Furthermore, the combination of MCC and silver nanoparticles in PLA-based formulations offered promising and valid perspectives to realize multifunctional composites with improved mechanical properties and antibacterial effects, maintaining at the same time the optical transparency and disintegrability in composting conditions—properties of fundamental importance in food packaging applications. Specifically, the disintegrability in compost showed that MCC-based composites had the highest rate of degradation; this effect was induced by the hydrophilic nature of filler systems [141].

Fortunati et al. also investigated the effect of silver nanoparticles combined with cellulose nanocrystals (CNC) in PLA-based systems [142]: a twin-screw extrusion process was used to produce PLA-based nanocomposites prepared by the addition of cellulose nanocrystals (CNC), eventually modified with surfactant (s-CNC) (binary films) and silver nanoparticles (ternary films). The nanocomposite formulations showed good optical properties, specifically in terms of transparency. The thermal analysis revealed that the presence of CNC and mostly the addition of s-CNC in binary-based systems induced an increase in the value of crystallinity and the highest tensile Young’s modulus. The presence of surfactant improved the dispersion of cellulose nanocrystals in the polymeric matrix and the nucleation effect was enhanced. Moreover, the antimicrobial activity of ternary-based systems underlined an antibacterial response against *Staphylococcus aureus* and *Escherichia coli* cells, suggesting that these novel nanocomposites are good alternatives for the food packaging sector, which requires an antibacterial effect and good mechanical response [142].

In addition, the effect of modified cellulose nanocrystals (s-CNC) and Ag nanoparticles combined with PLA was analysed in terms of barrier properties and overall migration limit. The combination of s-CNC and AgNPs contributed to enhance the barrier properties of neat polymer and to maintain the overall migration level, in contact with food simulants of different systems, below the allowed limit [143].

Similar results regarding the effect of reinforcement phase of cellulosic nanostructures in polymer matrix were obtained with other cellulose nanostructures extracted from different natural sources and combined with different biodegradable matrices. Cellulose nanocrystal and cellulose nanofibres extracted from sunflower stalks were used in gluten-based nanocomposites, with the main aim of increasing the mechanical and barrier properties of neat polymer [144], while other examples are available for flax and phormium fibres [145] and barley derivatives [146] in PVA, or *Posidonia oceanica* in combination with ZnO nanoparticles [139,147]. Luzi and co-authors also proposed the study of poly(lactic acid) and poly(butylene succinate) blends and bionanocomposite films prepared by the solvent casting method loaded with two different amounts of cellulose nanocrystals (1 or 3 wt %) extracted by acid hydrolysis from Carmagnola carded hemp fibres [136]. In this study, the authors proposed the effect of unmodified (CNC) and surfactant-modified cellulose nanocrystals (s-CNC) in PLA matrix and in PLA/PBS-blend-based systems in terms of thermal, morphological, mechanical and barrier properties. Tensile testing showed the positive effect of CNC and s-CNC in PLA- and PLA/PBS-based systems, while the Young’s modulus increased in PLA and in PLA blend formulations loaded with CNC (Table 2), highlighting the reinforcement effect induced by unmodified cellulose nanocrystals but a decrease of elongation at break of CNC-based nanocomposites with respect to PLA film (a pronounced effect was registered for PLA_3CNC film). s-CNC induced in PLA-based nanocomposites a different behaviour; the elongation at break underlined the ductile effect due to the presence of surfactant-modified cellulose nanocrystals.

Luzi and co-authors observed an encouraging synergic effect of PBS and both cellulose nanocrystals (CNC and s-CNC) in terms of barrier properties. CNC and s-CNC reduced the oxygen (OP) and water vapour (WVP) permeability (Table 2) [136]. This effect was related to the presence of PBS, which increases the crystallinity degree, and to the ability of cellulosic nanofillers to increase the tortuous path of gas molecules. The data obtained from the test of disintegrability under composting conditions revealed that all the formulations disintegrated in less than 17 days, while the presence of cellulose nanocrystals modified with surfactant was able to facilitate the disintegration behaviour, although the PBS presence hinders the disintegrability [136]. The effect of CNC addition has been also recently investigated in combination with eco-friendly copolyesters containing butylene succinate (BS) and triethylene succinate (TES) sequences [39]. The effect of TES and CNC presence and content on the microstructure, tensile properties, thermal characteristics and disintegration under composting conditions as well as on the toughening mechanism of extruded blend, was investigated. The results showed how, by combining two different strategies, i.e., copolymerization and nanofiller addition, it is possible to produce thin extruded films with a tunable range of properties by varying the amount and comonomeric unit (TES sequences) and/or of the nanofiller (s-CNC), confirming how the combination of synthesized biodegradable polymers with bio-based nanostructures allowed for the development of advanced functional materials capable of meeting the requirements for a wide range of applications.

Armentano and co-authors investigated the effect of plasticizer (lactic acid oligomer, OLA) at three different concentrations (15, 20 and 30 wt % by weight) in the polymeric blend of poly(lactic acid) (PLA) and poly(3-hydroxybutyrate) (PHB) (15 wt % with respect to PLA). The different formulations were realized by an extrusion process to improve the processability and mechanical characteristics of the biopolymers [59]. OLA acted as a plasticizer in PLA_15PHB-based formulations, as observed by the decrease in glass transition temperatures and the increase of ductile behaviour. The improvement of barrier properties of blend films is related to the higher crystallinity of PLA_15PHB-based formulations; the overall migration test underlined that all the formulations maintained the level of migrated substance of polymeric films in contact with food simulants below the admitted limit level [59]. PLA_15PHB film plasticized with OLA (15 wt %) was combined with 10 wt % of carvacrol used as an antioxidant agent. The ternary system with carvacrol maintained the mechanical response of the PLA_15PHB blend, while the addition of OLA induced a reduction of the Young’s modulus with an increase in the elongation at break. The presence of carvacrol in PLA_15PHB-based films improved the antioxidant response of different systems. The different formulations showed interesting mechanical and antioxidant properties for the food packaging sector [59].

**Figure 3:**
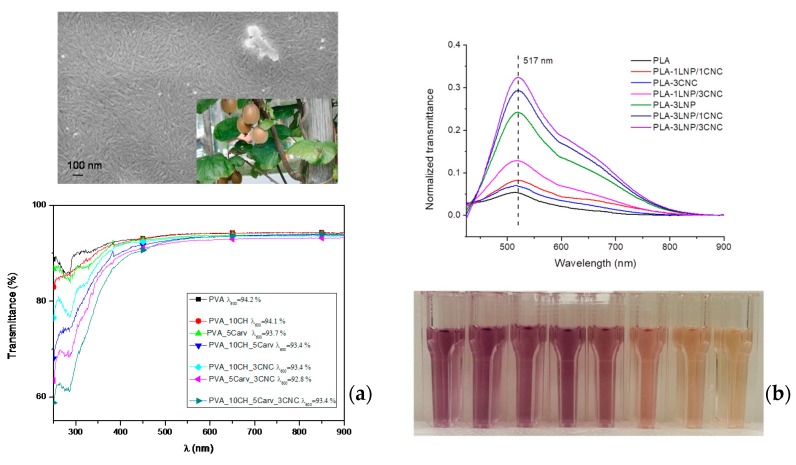
(**a**) FESEM investigation of CNC extracted from pre-treated kiwi fibres and UV–vis analysis of PVA-based formulation; (**b**) antioxidant activities of migrating substances for different PLA nanocomposite films immersed directly in the methanol solution for 24 h: monitoring of the absorbance for band at 517 nm and colour variation of the DPPH methanol solution (reprinted with permission from [137,148,149]).

Recently, Luzi and co-authors proposed the extraction of cellulose nanocrystals from kiwi *Actinidia deliciosa* pruning residues and their use as nanoreinforcement phases in poly(vinyl alcohol) (PVA) blended with chitosan (CH)-based systems, even in combination with carvacrol, used here as the active ingredient [137]. In this paper, we proved that the presence of CNC and carvacrol did not induce particular alterations in PVA and PVA_CH in cryo-fractured surfaces. However, the typical nucleation phenomenon induced by the presence of cellulose nanocrystals in thermoplastic polymers was not so evident when CNCs were used as the nanofiller phase in PVA_CH; this behaviour is related to the presence of chitosan, with its amorphous nature. We realized that the proposed formulations had significant antioxidant activity (Figure 3a), confirming how the presence of carvacrol could positively tune this property, which, along with transparency, is necessary for solvent-cast polymeric systems to be used in the food packaging sector. The presence of carvacrol also induced antimicrobial activity with respect to *Pectobacterium carotovorum* susp. Odoriferum (Pco) and *Xanthomonas arboricola* pv. Pruni (Xav). Pco and Xav bacteria/multiplication was reduced by using PVA_5Carv_3CNC after 12 h of testing [137].

This antioxidant activity was obtained for food packaging purposes even when bio-based nanoparticles, such as lignin-based nanoparticles, have been considered active fillers in extruded biopolymeric matrices, in combination (or not) with cellulose-based nano-reinforcements. In Yang et al. [149], the antioxidant and antimicrobial activities of lignin nanoparticles have been preliminary studied for potential biological applications, revealing how LNPs can effectively be used as active agents towards different pathogen strains. It was assumed that there was a relationship between antibacterial activity and the antioxidant property of lignin from the point of view of chemical structure, since the free-radical scavenging activity of lignin was found to be strictly related to the presence of phenolic structures on oxygen-containing reactive free radicals [150]. The authors investigated the role of the produced LNPs when they are incorporated in different polymeric films for packaging purposes (Figure 3b): two different PLA blends, one containing 3 wt % LNPs and one containing 3 wt % LNPs and 1 wt % CNCs, were tested against the bacterial plant pathogen *Pseudomonas syringae* pv. tomato (Pst) [148,151], while in another study the activity of neat PVA, CH, PVA/CH films and nanocomposites containing LNPs [152] was evaluated against Gram-positive and Gram-negative bacterial plant pathogens. The results revealed how all LNP-incorporated polymer blends showed reduced CFU concentrations within the first 3 h of contact, and remained at lower CFU concentrations when compared to the neat polymers, even after 24 h. Other than neat PLA and PVA/chitosan blends, Yang et al. also studied the effects of LNPs on the mechanical and thermal performance of wheat gluten (WG) and glycidyl methacrylate-grafted polylactic acid (g-PLA) [153,154]. In the case of water-soluble matrices, i.e., gluten and PVA, films were prepared with a casting method and aqueous LNP dispersions (1 and 3 wt %): the results confirmed a general decrease of light transmittance in the visible light spectrum after incorporation of LNPs (from 89% for neat WG to 56% for WG containing 3 wt % LNPs), while in the UV range (below 400 nm) transmittance below 2% was reached for the LNP containing PVA and WG samples.

Binary and ternary polymeric cast films with PVA, CH and LNPs were also prepared and the UV transmittance was reduced from 90.79% to quite low values (0.39% for PVA containing 3 wt % and 0.87% for ternary compositions) [152]. PLA/lignin nanoparticles films were also produced by extrusion and we observed, in the case of grafted PLA and 1 wt % LNPs, a 28% reduction of UV-B (280–315 nm) irradiation [154].

Briefly, we aimed to establish that the growth of nanotechnological approaches naturally supports the realization of nanocomposites containing these bio-based nanofillers; in addition, the processing approach could affect ultimate properties of polymeric matrices, having different rheological behaviour due a different effect of microstructure on manufacturing techniques parameters.

It should be recognized that the rheological characterisations of these materials, mostly orientated to better understanding the dynamics of confined polymers, can effectively support the investigation of many different items, such as degree of dispersion (intercalation and/or exfoliation), polymer–filler interaction and filler content, that are expected to be determining factors for the rheological response of nanocomposites. Rheological behaviour of polymer nanocomposites is affected by the discontinuity of material properties in the material domains, the presence of concentration gradient due to nonhomogeneity, and orientation of the flow element due to the presence of dispersed phases [155].

Rheological measurements at low deformation rates provide the most sensitive method for nanocomposite characterisation; moreover, the effect of all the variables previously mentioned can be considered in several simple rheological parameters. These concepts emphasise how rheology could be a promising and practical method to gain some control over nanocomposite technology. In particular, for polymer nanocomposites, the rheological properties are influenced by the nature of the structure formed, depending on the state of nanofillers’ dispersion in the matrix, the polymer–nanofiller interactions, imposed stress, and orientation of flow domains. From a rheological point of view, a direct consequence of the incorporation of fillers in polymers is a significant change in their steady shear viscosity behaviour and the viscoelastic properties [156]. In biopolymeric nanocomposites, measurements of melt rheological properties are not only important to understand the nature of processability of these materials, but also to determine the strength of polymer–filler interactions and the structure–property relationship in the nanocomposites, because melt rheological behaviours are strongly influenced by their nanoscale structure and interfacial characteristics. The evaluation of rheological behaviour for a series of different biopolymers was considered by Bari et al. [6]; in that paper, the authors reviewed recent studies carried out on biodegradable polymer nanocomposites, including preparation, characterization (comprising rheology), properties, and applications of nanocomposites based on biodegradable polymers and a wide range of organic and inorganic nanoparticles used as additives. Other than this, the interpretation of rheological behaviour for every matrix/filler combination could be extremely difficult or, on the other hand, too generic if not deeply investigated.

## 7. Conclusions and Outlook

The processing of novel biomaterials will create new challenges in the field of plastics engineering, and set new requirements for equipment, optimization and control. Currently, a significant amount of work is being published on nanocomposites based on biodegradable polymers. In an attempt to further understand the synthesis, processing and properties analysis of polymer nanocomposites, a selection of representative and recent literature was chosen to highlight some of the issues related to the preparation and mechanical behaviour of composites with nano-sized reinforcement in comparison with composites with larger micron-sized inclusions. The development of nanocomposites opens up new challenges in the field of biomedicine, yielding biocompatible materials with enhanced and modulated bulk and surface properties. We hope that our research activities in this field can open up new perspectives for their application, and help solve some technological problems.

## Figures and Tables

**Table 1:** Examples of the specific biological applications achieved using different types of stem cells and biomaterials, in terms of polymers, nanocomposites and gels.

Stem Cells	Biomaterials	Applications	Ref.
Embryonic stem cells			
	Nanostructured poly(d,l-lactide-*co*-glycolide)/collagen scaffolds	cardiomyocytes	[89]
	alginate microcapsules	midbrain dopamine neurons	[90]
	PNIPAAm-PEG thermoresponsive hydrogel	Oligodendrocyte precursor cells	[91]
	biocompatible and elastic polydimethylsiloxane (PDMS) scaffolds	differentiation of chondrogenic cells	[92]
	6-O-sulfated chitosan	neural differentiation	[93]
Adult stem cells			
Adipose stem cells	Methacrylated gelatine (GelMA) and methacrylated hyaluronic acid (HAMA)	promote angiogenesis for wound healing	[94]
	Strontium-hardystonite-Gahnite	osteogenic differentiation	[95]
	Polymeric INTEGRA^®^ Meshed Bilayer Wound Matrix	osteogenic differentiation adipogenic differentiation	[96]
	Poly(lactic-*co*-glycolic-acid/polyethylene glycol microspheres containing vascular endothelial growth factor in collagen-chitosan scaffolds	supported by a predominant vascular vessel	[97]
Bone marrow mesenchymal stem cells	Alginate hydrogel	axonal growth	[98]
	Polydopamine-laced hydroxyapatite collagen calcium silicate	Osteogenic differentiation	[99]
	Hyaluronic acid silk fibroin-poly(ε-caprolactone	Cardiomyocytes differentiation	[100]
	Silk fibroin films decorated with integrin-binding laminin peptide motifs (YIGSR and GYIGSR) in the presence of a biochemical cue	neuron-like cells	[101]
	Hydrogenated amorphous carbon (a-C:H) groove topographies in the absence of a biochemical cue	Neuron-like differentiated cells	[102,103]
Neural stem progenitor cells	gel-like (SLIDING) fibres	proliferation and neurosphere formation within the fibrous structures without compromising viability.	[104]
	Immobilization of poly-l-lysine and fibronectin on poly(lactic-*co*-glycolic acid)	Neuronal differentiation	[105]
	Hierarchically patterned substrate	Functional neurones	[106]
Induced pluripotent stem cells	Gelatine-poly(lactide-*co*-glycolide) nanoparticle	Pancreatic differentiation	[107]
	photo-cross-linked chitosan hydrogels	Neuronal differentiation	[108]
	polycaprolactone (PCL)/gelatine scaffolds	chondrogenesis	[109]
	mineralized gelatine methacrylate-based matrices	Osteogenic differentiation	[110]
	fibrous mats of poly(l-lactic acid) (PLLA) loaded with 1 or 8 wt % of calcium-deficient nanohydroxyapatite (d-HAp)	Osteogenic differentiation	[37]

**Table 2:** Mechanical properties and barrier properties of PLA- and PLA/PBS-based systems. Reprinted from [136].

*Formulation*s	Mechanical Properties			Barrier Properties	
	σ_b_ (MPa)	ε_b_ (%)	E_Young_ (MPa)	OP 10^12^ (cm^3^ m^−1^ s^−1^ Pa^−1^)	WVP (100–53% RH) (g mmkPa^−1^ h^−1^ m^−2^)
PLA	32.7 ± 6.3	330 ± 50	1250 ± 190	1.98 ± 0.08	0.079 ± 0.004
PLA_1CNC	26.8 ± 2.5	275 ± 15	1300 ± 50	1.66 ± 0.06	0.073 ± 0.003
PLA_3CNC	22.7 ± 3.2	160 ± 30	1540 ± 60	1.57 ± 0.05	0.087 ± 0.013
PLA_1s-CNC	22.4 ± 3.9	300 ± 30	1400 ± 100	1.61 ± 0.05	0.087 ± 0.013
PLA_3s-CNC	28.0 ± 4.8	270 ± 20	1260 ± 75	1.49 ± 0.04	0.068 ± 0.002
PLA_20PBS	30 ± 5.0	360 ± 30	920 ± 30	1.35 ± 0.02	0.065 ± 0.003
PLA_20PBS_1CNC	15.5 ± 2.2	210 ± 10	950 ± 50	1.26 ± 0.01	0.063 ± 0.003
PLA_20PBS_3CNC	21.4 ± 3.3	230 ± 25	1130 ± 90	1.09 ± 0.04	0.071 ± 0.008
PLA_20PBS_1s-CNC	23.5 ± 4.1	260 ± 25	970 ± 20	1.19 ± 0.02	0.055 ± 0.005
PLA_20PBS_3s-CNC	20.1 ± 1.6	370 ± 40	1120 ± 75	1.05 ± 0.02	0.062 ± 0.002
